# Aetiological relevance of haematological, biochemical and endocrine parameters on equine odontoclastic tooth resorption and hypercementosis (EOTRH)

**DOI:** 10.1111/evj.14555

**Published:** 2025-07-08

**Authors:** Melusine Tretow, Anna M. Hain, Astrid Bienert‐Zeit

**Affiliations:** ^1^ Clinic for Horses University of Veterinary Medicine Hannover, Foundation Hannover Germany

**Keywords:** dental, horse, hypovitaminosis D, pathogenesis, selenium deficiency

## Abstract

**Background:**

The dental syndrome EOTRH is a painful, progressive dental disease with an unknown aetiology. The often painful nature of EOTRH emphasises the need for a better knowledge of the underlying pathogenic mechanism and risk factors. A comparative analysis of haematological, biochemical and endocrine values in EOTRH‐affected and non‐affected horses has not been described.

**Objectives:**

To compare haematological, biochemical, and endocrine parameters in EOTRH‐affected and non‐affected horses to detect risk factors for horses developing EOTRH.

**Study Design:**

Cross sectional.

**Methods:**

Blood samples of 154 Icelandic horses aged 15 years and older were collected. A CBC, biochemistry panel, and endocrine profile were performed. A detailed examination of the rostral oral cavity was performed, and incisors were evaluated radiographically using a standardised scoring system. Based on the results, the study population was separated into ‘EOTRH‐affected’ (*n* = 109) and ‘EOTRH‐nonaffected’ (*n* = 23) horses. A staging system enabled further differentiation into mild (Stage 2), moderate (Stage 3) or severe (Stage 4) EOTRH‐affected versus Stage 0 (EOTRH‐nonaffected). To assess the correlations between EOTRH diagnosis and the measured parameters, logistic regression analysis was performed.

**Results:**

No consistent abnormalities were detected in the CBC. In the biochemistry panel, selenium deficiency (38%) and hypovitaminosis D (83%) were the only consistent abnormalities observed across the entire study population. Endocrine analytes showed no abnormalities in thyroid function. Pituitary pars intermedia dysfunction was diagnosed in 17% of the horses.

**Main Limitations:**

Irregular distribution of horses between the control group and the EOTRH‐affected group. Plasma concentrations were measured only once, and no functional tests of the thyroid gland, nor an oral sugar test or TRH stimulation test were performed.

**Conclusions:**

EOTRH triggers a predominantly local inflammation in the oral cavity, without measurable changes in the inflammatory cells or significant variations in plasma vitamin and trace element serum concentrations.

**Abbreviations**
ACTH: adrenocorticotropic hormone
BCS: body condition score
Ca: calcium
CBC: complete blood count
CLIA: chemiluminescence immunoassay
CNS: cresty neck score
EIA: enzyme immunoassay
EOTRH: equine odontoclastic tooth resorption and hypercementosis
ERY: erythrocytes
fT4: free thyroxine
Hct: haematocrit
Hgb: haemoglobin
HPLC/U‐HPLC: (ultra‐) high performance liquid chromatography
ICP‐MS: inductively coupled plasma mass spectrometry
LEU: leucocytes
MCH: mean corpuscular haemoglobin
MCHC: mean corpuscular haemoglobin concentration
MCV: mean corpuscular volume
P: inorganic phosphate
Se: selenium
T3: total triiodothyronine
T4: total thyroxine 4
TC: thrombocytes
Vit A: vitamin A
Vit D3: 25‐hydroxy‐vitamin‐D3
Zn: zinc

## 1 INTRODUCTION

Equine odontoclastic tooth resorption and hypercementosis (EOTRH) is a potentially painful, progressive dental disease mainly affecting the incisor and canine teeth, and less frequently, the cheek teeth, of senior‐aged horses.^1^, ^2^, ^3^ The symptoms of EOTRH can vary from nearly asymptomatic to a severely impaired general condition and behaviour. Non‐age correlating bite angle of the incisors, subgingival swelling, gingival recession, fistulae and gingivitis are indicative of the presence of EOTRH.^4^ Aside from clinical findings, EOTRH diagnosis is based on radiographic findings like varying degrees of tooth resorption and bulbous enlargement of the intraalveolar tooth aspects. This is due to the histopathological mechanisms of EOTRH, consisting of chronological sequences of odontoclastic resorption followed by reparative cementogenesis.^5^ Other than clinical and histological features, the aetiology as well as the pathological process of EOTRH are unknown. High age and the male sex are known risk factors for horses contracting EOTRH.^6^ Furthermore, Icelandic horses seem to be more prone to EOTRH.^6^, ^7^

In addition to genetic predisposition, biomechanical stress of the periodontal ligament^5^ and bacterial genesis are considered possible hypotheses for the aetiology of EOTRH.^8^ Metabolic and endocrine disorders, such as pituitary pars intermedia dysfunction (PPID), equine metabolic syndrome (EMS) and nutritional secondary hyperparathyroidism, may have an influence.^9^ Further theories suggest that deficiencies or excesses in vitamins and trace elements, such as calcium and zinc, which are both essential for tooth mineralisation,^10^ could play a role. So far there has been no comparison of haematological, biochemical and endocrine parameters of EOTRH‐affected and non‐affected horses.

Currently, the removal of affected teeth is the only long‐term solution to alleviate the pain associated with EOTRH in horses. Therefore, it is highly desirable to develop improved treatment and prevention methods, which requires a deeper understanding of the disease's aetiology. The aim of this study was to contribute to this knowledge. Specifically, the study sought to characterise differences in blood parameters between EOTRH‐affected and non‐affected horses. We hypothesised that (a) EOTRH often occurs simultaneously with PPID and EMS, (b) plasma selenium and zinc concentrations differ between EOTRH‐affected and non‐affected horses and (c) EOTRH‐affected horses show increased leucocyte counts.

## 2 MATERIALS AND METHODS

### 2.1 Study population

Only pure‐bred Icelandic horses aged 15 years and older with at least one remaining incisor were included in the study. The horse population consisted of 64 mares and 90 geldings or stallions ranging in age from 15 to 31 years, with an average age of 20.1 years. Horses were recruited randomly within Lower Saxony, Germany, and the investigations took place on an outpatient basis.

The horses' previous medical history was obtained through a standardised questionnaire completed by the owners (Data S1) covering various aspects, including animal husbandry practices and feeding, such as the provision of concentrated feed and supplements like selenium (Se). Additionally, information on diseases such as EOTRH, PPID and EMS was acquired from owners.

To ensure that only horses in good general condition were included, all horses underwent a clinical examination, and exclusion criteria were established (Data S2). The clinical examination also included the assessment of the body condition score (BCS) and the cresty neck score (CNS).^11^, ^12^

### 2.2 Blood sample collection

The horses were not given any concentrated feed within 6 h before sample collection, while roughage and water intake were not restricted. Blood samples were usually collected from the left superficial jugular vein using the BD Vacutainer‐System (Becton, Dickinson and Company). EDTA plasma was obtained by centrifugation immediately after collection, then chilled and shipped frozen. The serum tubes were stored in the dark and centrifuged after a clotting time of 30 min, before being shipped refrigerated. Additionally, a serum sample was stored at −80°C for later dispatch to the laboratory. A blood smear was also prepared from EDTA blood. All samples were sent to the same diagnostic laboratory (IDEXX laboratories, Vet Med Labor GmbH, Kornwestheim, Germany).

### 2.3 Blood tests

Blood tests included a complete blood count (CBC) without differential, as well as biochemical and endocrine parameters.

The CBC parameters included leucocytes (LEU), erythrocytes (ERY), haemoglobin (Hgb), haematocrit (Hct), mean corpuscular volume (MCV), mean corpuscular haemoglobin (MCH), mean corpuscular haemoglobin concentration (MCHC) and thrombocytes (TC). Biochemical parameters measured were calcium (Ca), inorganic phosphate (P), glucose, selenium (Se), zinc (Zn), vitamin A (Vit A) and 25‐hydroxy‐vitamin D3 (Vit D3). To minimise calibration‐related fluctuations in measured values for Zn, Se, Vit A, and Vit D3, additional serum samples for these parameters were stored at −80°C and analysed simultaneously. Endocrine parameters measured included adrenocorticotropic hormone (ACTH), insulin, total thyroxine 4 (T4), free thyroxine (fT4) and total triiodothyronine (T3).

### 2.4 Clinical and radiographic examination of the oral cavity

Further examinations were conducted on horses sedated with an α‐2 adrenergic agonist‐opioid combination (detomidine [Cepesedan, cp‐Pharma], 0.012–0.03 mg/kg, IV, once and butorphanol [Butorgesic, cp‐Pharma], 0.025–0.04 mg/kg, IV, once). After evaluating the clinical appearance of the rostral oral cavity using a standardised protocol (Table S1), digital intraoral radiography of the incisors was performed. A minimum of two intraoral radiographs were taken per horse: one of the maxilla (0°/+45°) and one of the mandible (0°/−80°). Three veterinarians assessed the X‐ray images using a standardised scoring system including number of affected or missing teeth, tooth shape, tooth structure and tooth surface (Table S2). The median score was calculated from the total scores provided by all three observers and was transferred to an adjusted radiological staging system based on Hüls et al.^13^ and Rehrl et al.^14^ This system classified horses into five X‐ray stages: ‘No EOTRH’ (Stage 0), ‘Suspicious’ (Stage 1), ‘Mild’ (Stage 2), ‘Moderate’ (Stage 3) or ‘Severe’ (Stage 4) (Table 1).

**TABLE 1:** Adjusted radiological staging system (based on Hüls et al.^13^ and Rehrl et al.^14^).

	Stage	Score	
No EOTRH	0	0	No radiological findings
Suspicious	1	1–2	Tooth shape preserved, but sporadic deviations: slightly blunted root tip, surface irregular/rough, slightly altered tooth structure
Mild	2	3–5	Tooth shape preserved, slightly blunted root tip, surface irregular/rough, slightly altered tooth structure
Moderate	3	6–9	Tooth shape largely preserved, intraalveolar tooth part is not wider than the clinical crown, obviously blunted root tip, surface irregular/rough, moderately altered tooth structure
Severe	4	>10	Loss of tooth shape, intraalveolar tooth part wider than the clinical crown, surface obviously irregular/rough, severely altered tooth structure

*Note*: The radiographs were evaluated independently by three veterinarians using a standardised scoring system. The median was calculated from the total scores of all three observers and used to classify them into Stages 0–4.

### 2.5 Data analysis

#### 2.5.1 Sample size calculation and statistical analysis

The initial sample size calculation was performed using PASS 2019, version 19.0.3, with Mixed Model Tests for Two Proportions in a 2‐Level Hierarchical Design (Level‐1 Randomisation). A significance level (*α*) of 5% and a statistical power of 80% were chosen based on standard clinical research practices to minimise Type I and Type II errors while ensuring the detection of clinically meaningful differences.

The calculation assumed that ~20% of animals in the study population would be affected by PPID, a figure supported by existing literature. Additionally, it was anticipated that EOTRH would occur 30 percentage points more frequently in animals with PPID than in those without it, which was considered a clinically significant difference. The worst‐case scenario was considered to account for variability in the prevalence of EOTRH in the non‐PPID group. This scenario ensures that the sample size would be adequate to detect the expected effect, even under highly variable conditions.

The null hypothesis (H_0_) posits no difference in the prevalence of EOTRH between the two groups, while the alternative hypothesis (H_1_) posits that the prevalence differs. Based on these assumptions, the sample size calculation indicated that 150 animals would be required to achieve 80% power at a 5% significance level.

#### 2.5.2 Descriptive statistics and categorisation of ACTH and insulin

Descriptive statistics for each blood parameter were calculated using Microsoft Excel (Version 2411 Build 16.0.18227.20082, Microsoft Corp.). The mean, median, minimum and maximum values were determined, and frequency distributions of values within and outside the normal range were reported as percentages or absolute numbers. For season‐dependent parameters (ACTH, Vit D3), the time of year was taken into consideration.

ACTH and insulin concentrations were classified according to established reference ranges into the following categories: ‘positive’, ‘negative’, ‘suspicious’ and ‘treated’ (for ACTH only). The ‘suspicious’ category indicated borderline results, while the ‘treated’ category referred to horses that had previously received treatment for PPID.

For classifying horses as PPID‐affected or non‐PPID‐affected, ACTH concentrations were evaluated in accordance with the Recommendations for the Diagnosis and Treatment of PPID (Equine Endocrinology Group, 2017). Horses with ACTH concentrations exceeding the established threshold were classified as PPID‐positive, while horses with normal ACTH concentrations and no prior history of PPID treatment, according to their medical records, were classified as PPID‐negative.

#### 2.5.3 Statistical tests and regression analyses

The association between EOTRH status (Stage 0 and Stages 2–4) and PPID was tested using an exact *χ* ^2^‐test. Horses in Stage 1 (suspicious results) were excluded from the logistic regression analysis due to ambiguous diagnosis. To explore potential differences in the proportion of values within the normal range between EOTRH‐affected (Stages 2–4) and non‐affected (Stage 0) horses, logistic regression was performed for the following binary dependent variables: CBC parameters, ACTH, insulin, glucose, Se, Zn, Vit A, Vit D3, T3, T4 and fT4. These dependent variables were categorised as either ‘within the normal range’ or ‘outside the normal range’ based on established reference values. Logistic regression was used to examine whether EOTRH status (affected/non‐affected) influences the probability of values outside the normal range for each parameter. EOTRH status was used as the independent variable. A *p*‐value of <0.05 was considered statistically significant.

#### 2.5.4 Exploratory nature of the study

It is important to note that this study is exploratory in nature. Consequently, while the findings offer valuable insights, they should be interpreted with caution, especially as no adjustments for multiple comparisons were made. The family‐wise error rate (i.e., the probability to make at least one type I error) might be large due to the large number of comparisons performed. The results should be regarded as preliminary and hypothesis‐generating, rather than definitive. Further studies with more rigorous statistical controls, including corrections for multiple comparisons, are necessary to confirm these associations and provide more robust conclusions.

#### 2.5.5 Software for statistical analysis

Statistical analyses were performed using SAS‐EG (version 7.15) and SAS Software (version 9.4, SAS Institute Inc.).

## 3 RESULTS

Data from 151 of 154 horses met the inclusion criteria for further evaluation. The findings of three horses (P95, P143, P147) had to be excluded due to lack of X‐rays. Overall, 72.2% (*n* = 109) of horses were diagnosed with EOTRH (Stage ≥2), while 15.2% (*n* = 23) showed no radiological findings (Stage 0). A total of 12.6% (*n* = 19) of the horses were staged as ‘suspicious’ (Stage 1). Among the EOTRH‐affected horses, 17.2% (*n* = 26) were mildly affected (Stage 2), 29.2% (*n* = 44) were moderately affected (Stage 3) and 25.8% (*n* = 39) were severely affected (Stage 4).

### 3.1 Complete blood count without differential

The percentages of horses with CBC values either above or below the normal range are shown in Table 2. CBC results were not available for three horses. Overall, for most of the CBC parameters, less than 30% of the values were not within the normal range (Table 2). A higher percentage of deviations was found in TC counts only (37%, Table 2). Only one horse diagnosed with EORTH had leucocytosis (19.6 G/L, X‐ray Stage 4). This horse was suffering from chronic lymphocytic leukaemia. In one horse, there was a high degree of platelet aggregation, which made it impossible to count the platelets; in this case, TC = 0 was assumed. Logistic regressions showed no statistically significant effect of EOTRH status on blood count values being in the reference interval (*p* between 0.14 for TC and 0.96 for MCH and MCHC).

**TABLE 2:** Complete blood count and percentage distribution of abnormal values above and below normal range for non‐affected (Stage 0) and EOTRH‐affected (Stage ≥2, mildly to severely affected) horses.

Parameter	*n*	Median (min–max)	% normal	% above normal range		% below normal range		Normal range
				Stage 0	Stage ≥2	Stage 0	Stage ≥2	
LEU (G/L)	148	6.7 (3.9–19.6)	95.9%	0.7%	0.7%	0.7%	1.4%	4.3–10.7
ERY (T/L)	148	6.8 (4.6–9.1)	73.6%	0.0%	0.0%	2.7%	20.3%	6.3–10
Hgb (g/dL)	148	11.3 (1.8–14.7)	71.6%	0.0%	0.0%	3.4%	19.6%	10.7–16.3
Hct (%)	148	31.5 (22.2–39.1)	79.1%	0.0%	0.0%	2.0%	13.5%	29.0–42.4
MCV (fL)	148	46.5 (40.6–55.7)	97.3%	0.0%	2.0%	0.0%	0.0%	39.5–51.5
MCH (pg)	148	16.8 (14.7–19.2)	98.6%	0.7%	0.0%	0.0%	0.0%	14.6–19.1
MCHC (g/dL)	148	36.2 (33.8–38.6)	83.1%	0.0%	0.0%	2.7%	10.8%	35.4–40.3
TC (G/L)	147	173 (0–612)	62.8%	6.8%	24.3%	1.4%	1.4%	64–195

Abbreviations: ERY, erythrocytes; Hct, haematocrit; Hgb, haemoglobin; LEU, leucocytes; MCH, mean corpuscular haemoglobin; MCHC, mean corpuscular haemoglobin concentration; MCV, mean corpuscular volume; *n*, number of horses with test results; TC, thrombocytes.

### 3.2 Biochemical parameters

The plasma concentrations of the biochemical analytes are presented in Table 3. Plasma Ca and P concentrations of Stage 0 and Stage ≥2 animals were inside the normal range (normal range). Deviations from the normal range in plasma concentrations of Vit A and Zn were found in less than 10% of all horses. Decreased plasma Se concentrations were measured in 37.4% (*n* = 40) of the horses with EOTRH and in 21.7% (*n* = 5) of the ‘EOTRH‐nonaffected’ horses. An increased plasma Se concentration was measured in only 2 of the 149 horses. The greatest divergences from the normal range were observed for plasma Vit D3 concentrations. Both within stage 0 (*n*_total_ = 23, *n*_belowNR_ = 19) and stages ≥2 (*n*_total_ = 107, *n*_belowNR_ = 89), over 80% of the horses had reduced plasma Vit D3 concentrations. Samples collected during the summer months (June to August, *n*_summer_ = 38) showed that 71% (*n*_belowNR_ = 27) of the horses had plasma Vit D concentration below the normal range. In samples collected in winter (*n*_winter_ = 92), 88% (*n*_belowNR_ = 81) of the horses were measured with a plasma Vit D concentration below normal range.

**TABLE 3:** Biochemical parameters and percentage distribution of abnormal values above and below normal range for EOTRH‐affected (Stage ≥2) and non‐affected horses (Stage 0).

Parameter	*n*	Median (min–max)	% normal	% above normal range		% below normal range		Normal range
				Stage 0	Stage ≥2	Stage 0	Stage ≥2	
Zn (μg/L)	149	475 (291–754)	92.6%	0%	0%	0.7%	4.7%	400–1100
Se (μg/L)	149	87 (11–445)	61.8%	0.7%	0.7%	3.4%	26.8%	70–170
Vit A (mg/L)	149	0.13 (0.008–0.22)	94.6%	0%	0%	0.7%	3.4%	0.1–0.37
Vit D3 (nmol/L)^a^	149	3.5 (2.9–14)	16.8%	0%	0%	12.8%	59.7%	5.2–18.2
Ca (mmol/L)	149	3.1 (2–3.4)	99.3%	0%	0%	0%	0%	2.3–3.4
P (mmol/L)	150	0.9 (0.5–1.6)	100%	0%	0%	0%	0%	0.4–1.7

Abbreviations: Ca, calcium; P, inorganic phosphate; Se, selenium; Vit A, vitamin A; Vit D3, 25‐hydroxy‐vitamin‐D3; Zn, zinc.

^a^ Values below 3 nmol/L were not measured more precisely. For the calculation of the median, 2.9 nmol/L was assumed.

Overall, no statistically significant differences were noted between EOTRH‐affected and non‐affected horses concerning the proportion of animals in the normal range (*p* between 0.2 for Zn and 0.9 for Vit A).

### 3.3 Endocrine analytes

An overview of the thyroid, insulin and ACTH concentrations is provided in Table 4. Only T4 showed a significant influence of EOTRH status on the proportion of observations within normal range (*p* = 0.03). The proportion of T4 values outside the normal range was smaller for EORTH‐affected animals (2 of 109) compared with non‐affected animals (3 of 23). No significant effect was found for fT4, T3 and glucose (*p* values ranged from 0.5 for fT4 to 0.9 for glucose).

**TABLE 4:** Endocrine parameters and percentage distribution of abnormal values above and below normal range for EOTRH‐affected (Stage ≥2) and non‐affected horses (Stage 0).

Parameter	*n*	Median (min–max)	% normal	% above normal range		% below normal range		Normal range
				Stage 0	Stage ≥2	Stage 0	Stage ≥2	
T4 (μg/dL)	151	1.6 (0.7–3.9)	96.0%	0%	0%	2%	1.3%	1.0–3.8
fT4 (ng/dL)^a^	151	0.7 (0.29–1.8)	91.4%	0%	0.7%	2%	5.3%	0.5–1.6
T3 (μg/L)^b^	151	0.39 (0.186–3.01)	98.0%	0%	0%	0.7%	0.7%	0.2–1.8
Glucose (mg/dL)	151	90 (64–152)	86.8%	2%	9.9%	0%	0%	61–101
Insulin (mU/L)	151	3 (0.9–144)	95.4%	—	—	—	—	<20^c^
ACTH (pg/mL)	150	21.5 (3–715)	67.6%	—	—	—	—	—^d^

Abbreviations: fT4, free thyroxine; n, number of horses with test results; T3, triiodothyronine; T4, total thyroxine.

^a^ Values below 0.3 ng/dL were not measured more precisely. For calculation of the median, 0.29 ng/dL was assumed.

^b^ In seven cases only the value <0.4 μg/L was determined; in these cases 0.39 μg/L was assumed for the calculation.

^c^ Interpretation: <20 mU/L negative, 20–28 mU/L suspicious, >28 mU/L insulin dysregulation.

^d^ The evaluation was carried out according to Recommendations for the Diagnosis and Treatment of PPID. Equine Endocrinology Group 2017.

#### 3.3.1 Pituitary pars intermedia dysfunction (PPID)

**FIGURE 1:**
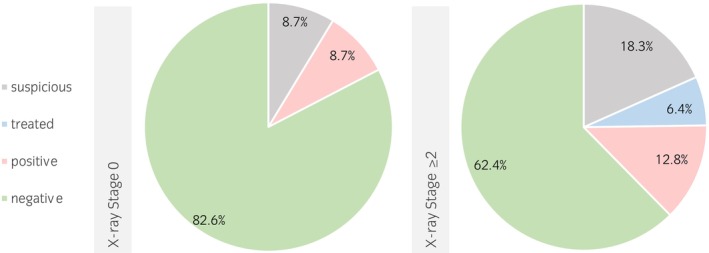
Percentage distribution of pituitary pars intermedia dysfunction (PPID) diagnosis for equine odontoclastic tooth resorption and hypercementosis (EOTRH)‐affected (Stage ≥2) and non‐affected horses (Stage 0).

Of the 150 horses examined, 67.3% (*n* = 101) had negative test results for PPID. Of the remaining horses, 16.6% (*n* = 25) were suffering from PPID. Among these, eight horses were already undergoing treatment, representing 5.3% of the total number of animals. Of the horses suffering from PPID, 84% (*n* = 21) also suffered from EOTRH (Stage ≥2), while 8% (*n* = 2) were staged as ‘EOTRH‐nonaffected’ (Stage 0). Among the PPID‐negative horses, 66% were suffering from EOTRH and 18.8% were in EOTRH Stage 0. The relative frequency of PPID in relation to the X‐ray staging is shown in Figure 1. PPID and EOTRH were not significantly associated (*p* = 0.2).

#### 3.3.2 Insulin dysregulation

In the entire study population, an insulin concentration >28 mU/L was measured in five horses. Two of these five horses were classified as ‘EOTRH‐non‐affected’ (Stage 0), while three were mildly to moderately EOTRH‐affected (Stage ≥2). Furthermore, one EOTRH‐nonaffected and one EOTRH diseased horse showed insulin values in the questionable range (20–28 mU/L).

#### 3.3.3 Body condition score (BCS) and cresty neck score (CNS)

**FIGURE 2:**
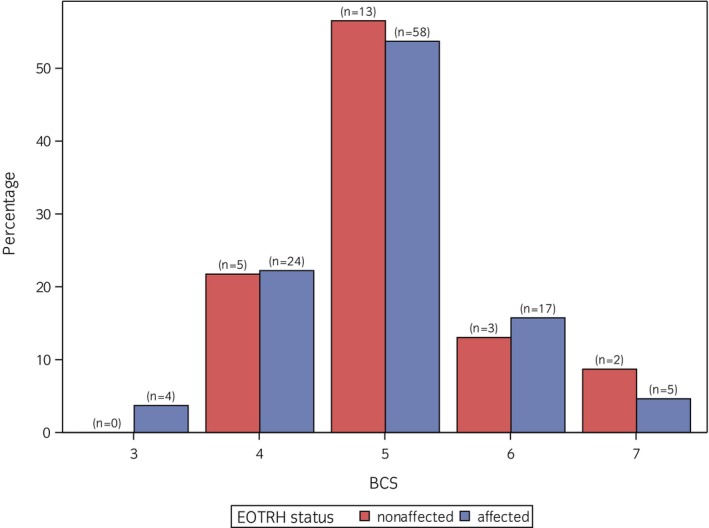
Distribution of body condition score (BCS) for equine odontoclastic tooth resorption and hypercementosis (EOTRH)‐affected and ‐nonaffected horses in the study population.

**FIGURE 3:**
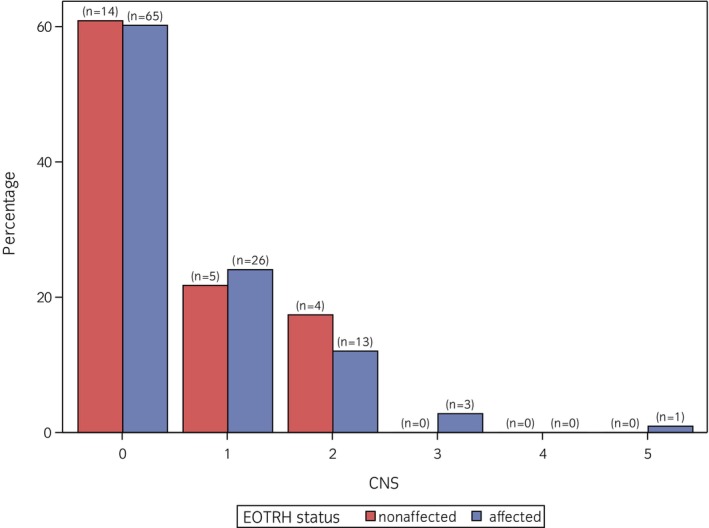
Distribution of CNS for equine odontoclastic tooth resorption and hypercementosis (EOTRH)‐affected and ‐nonaffected horses in the study population.

The distribution of BCS and CNS is visualised in Figures 2 and 3. The median BCS was 5, with a minimum of 3 and a maximum of 7. Within the ‘EOTRH‐nonaffected’ group, 21.7% (*n* = 5) had a BCS above 5, 8.7% (*n* = 2) had a BCS of 7. In the ‘EOTRH‐affected’ group, 20.4% (*n* = 22) had a BCS >5 and 4.6% (*n* = 5) even a BCS of 7. The distribution of BCS in both groups was quite similar. The median CNS was 0, with values ranging from 0 to 5. Among both EOTRH‐affected and non‐affected horses, ~39% of the horses had a CNS ≥1. Non‐affected horses did not score higher than 2, while in the affected group, three horses had a CNS of 3 and a CNS of 5.

## 4 DISCUSSION

Tooth resorption is a prevalent inflammatory oral disorder described in horses, cats,^15^ dogs^16^, ^17^ as well as humans.^18^ Nevertheless, the underlying aetiology remains unclear in all species. The current study is the first to compare haematological, biochemical and endocrine parameters of EOTRH‐affected horses with orally healthy horses.

As the leucocyte count remained relatively normal, there did not appear to be a haematological response to the different levels of local inflammation in the rostral oral cavity. A lack of a systemic inflammatory response indicates that in EOTRH the inflammatory reaction predominantly affects the oral cavity locally. Saliva of cats with feline odontoclastic resorptive lesions (FORL) has been reported to contain higher concentrations of pro‐inflammatory cytokines and chemokines as a result of local chronic inflammation, which are assumed to promote osteoclastic activity.^19^ Similar studies are still lacking for horses.

It is commonly known that PPID is associated with impaired immune function.^20^ Given the chronic inflammatory nature of EOTRH, it is reasonable to assume that PPID may exacerbate this inflammation. Although no statistically significant association was found between EOTRH and PPID in this study, Section 3.3.1 clearly indicates that most horses suffering from PPID also suffered from EOTRH and that the proportion of animals suffering from EOTRH in the PPID‐nonaffected animals was lower. This may be due to the fact that the incidence of both diseases increases with age.^6^, ^21^, ^22^ A direct link between the simultaneous occurrence of the two diseases cannot be confirmed or excluded.

Another metabolic disorder in horses is the equine metabolic syndrome (EMS), a collection of endocrine and metabolic abnormalities.^23^ EMS is associated with increased adiposity and insulin dysregulation, a collective term for hyperinsulinaemia and insulin resistance. In total, only five horses were diagnosed with insulin dysregulation in this study, and two others had values in a questionable range. Even though no further diagnostics such as an oral sugar test were carried out, it can be assumed that there was no aetiological link due to the low proportion of horses with hyperinsulinaemia and obesity compared with the high proportion of horses with EOTRH in the study population.

The results of the analysed plasma T3, T4 and fT4 concentrations indicated euthyroid conditions in the whole study population. The small deviations can be explained by the fact that in horses with PPID, lower serum concentrations of these analytes' values were described.^24^ A limitation of this study is that no functional test of the thyroid gland was carried out.

In addition to effects of endocrine disorders, the influence of nutrition as an aetiological factor for tooth resorption is also discussed. In cats, hypervitaminosis A is suspected to be associated with FORL due to the stimulating effect of retinol and tretinoin on clastic cells.^15^ Our investigations did not confirm this assumption for horses. In none of the horses was a hypervitaminosis A measured. This aligns with another study on EOTRH‐affected horses, which found plasma Vit A concentrations below the normal range in the majority of cases.^9^

Furthermore, microminerals are necessary for physiological body function. Selenium and zinc are essential components of antioxidant enzymes. With horses being relatively tolerable to high Zn supply, Zn deficiency can cause parakeratosis, erosions, ulcerations, and inflammation of the skin. Recent studies have shown that plasma Zn concentrations were widely unaffected by nutritional and non‐nutritional factors in horses and ponies.^25^ This is also consistent with the findings in this study, showing that plasma Zn concentration was within the normal range in almost all horses.

Se deficiency can induce oxidative stress, inflammation and abnormal autophagy.^26^, ^27^ Due to this important role in cell protection, plasma Se concentrations were measured. Se deficiency was detected in 37.4% of horse suffering from EOTRH, 52.6% of horses in Stage 1 (suspicious) and in 21.8% of ‘EOTRH‐nonaffected’ horses. Other studies also reported Se concentrations below normal range in 47%–49% of the healthy horses examined.^28^, ^29^ Mild variation above or below normal range does not necessarily appear to be clinically relevant, especially considering that Se concentrations can vary considerably depending on intake.^28^ Another point of limitation is the fact that Se concentrations were only measured once and no measurement of glutathione peroxidase was performed. A conclusion about the role of selenium supply in the development of EOTRH cannot be made based on a single measurement, as this does not allow any conclusions to be drawn about the supply at the time of the onset of the disease. This limitation can be transferred to the other measured trace elements and vitamins. Nevertheless, no statistically significant difference between the plasma Se concentrations of diseased and healthy horses could be determined for this study population.

Calcitriol (active Vit D), calcium and phosphorus are important for bone mineralisation due to influence (active Vit D) on parathyroid activity.^30^ Hypocalcaemia, hyperphosphatemia and low calcitriol concentrations favour parathyroid hormone (PTH) secretion. PTH stimulates indirectly via osteoblast osteoclastic bone resorption. Earley et al.^9^ suspected nutritional secondary hyperparathyroidism due to an inverse calcium: phosphorus ratio in concentrates and processed feed as the cause of EOTRH. This assumption was based on parathyroid hormone being above normal range in 47% of the horses. In the aforementioned study, 18 horses suffering from EOTRH were examined, including 30% fed concentrates as processed feed. In our study population, concentrates such as premixed horse muesli, cereals and mash were fed to 86.9% of the EOTRH‐affected horses and 95.7% of the EOTRH‐non‐affected horses. Horses received concentrates mostly occasionally and in small amounts. Daily feeding of concentrates occurred in <20% of cases.^6^ An analysis of concentrates for calcium, phosphorus and the presence of Ca‐binders was not carried out in either study. However, as almost all ‘EOTRH‐nonaffected’ horses in our study population were fed concentrates, it can be assumed that these concentrates, given in low amounts, are not the cause of nutritional secondary hyperparathyroidism.

The diagnosis of nutritional secondary hyperparathyroidism is based on a preliminary report, clinical, radiological, and biochemical findings. Clinical findings include lameness, weakness, ataxia and enlargement of the facial bones.^31^, ^32^ These findings do not align with those observed in the horses examined in our study. Laboratory testing in horses suffering from nutritional secondary hyperparathyroidism shows a decreased or normal plasma calcium concentration and increased phosphate.^31^ This contrasts with the fact that both Ca and P were within the reference range in the study population. It must be emphasised that no PTH was determined. Slightly reduced plasma Vit D concentrations (minimum = 2.9 nmol/L, normal range = 3.5–18.2 nmol/L) were found in 80% of both EOTRH‐affected and orally healthy horses. A. Hain's observations revealed that the majority of equines in Iceland had plasma Vit D concentrations below the normal range, as determined by her measurements. However, these findings have not yet been published.

Finally, it should be noted that this study population had an asymmetric distribution of orally healthy and EOTRH‐affected horses, which carries a potential risk of bias. This is due to the fact that horses initially presented as EOTRH‐nonaffected by the owner were diagnosed with EOTRH during the course of this study. On the one hand, this leads to an overrepresentation of horses with EOTRH; on the other hand, it emphasises how widespread and underdiagnosed EOTRH is within the German Icelandic horse population.

In conclusion, EOTRH induces a predominantly local inflammatory reaction in the oral cavity without any measurable changes in the inflammatory cells. Pro‐inflammatory cytokines in saliva, similar to those found in cats, or the immunodeficiency in PPID, may maintain this inflammatory response secondarily. Studies on the composition of equine saliva are still pending and have yet to confirm the involvement of such cytokines in horses. All in all, no statistically significant differences between EOTRH‐affected and ‐nonaffected horses in laboratory testing were found. No trace element appears to promote EOTRH due to an oversupply or undersupply. Further investigations by means of a follow‐up study monitoring the analysed parameters in a population until the onset of EOTRH would be necessary to reliably detect such influences.

## FUNDING INFORMATION

This work was supported by Stiftung Pro Pferd, Zurich, Switzerland [Grant number PR 2021‐01].

## CONFLICT OF INTEREST STATEMENT

The authors declare no conflict of interest.

## AUTHOR CONTRIBUTIONS

**Melusine Tretow:** Conceptualization; investigation; funding acquisition; writing – original draft; methodology; visualization; writing – review and editing; validation; project administration; data curation; resources; formal analysis. **Anna M. Hain:** Writing – review and editing. **Astrid Bienert‐Zeit:** Conceptualization; funding acquisition; methodology; validation; writing – review and editing; project administration; supervision; resources.

## DATA INTEGRITY STATEMENT

Melusine Tretow had full access to all the data in the study and takes responsibility for the integrity of the data and the accuracy of the data analysis.

## ETHICAL ANIMAL RESEARCH

The study was registered as a notifiable animal experiment under file number 33.8‐42502‐05‐20A552 at the Lower Saxony State Office for Consumer Protection and Food Safety (LAVES).

## INFORMED CONSENT

Owners gave consent for their animals' inclusion in the study.

## Supporting information


**Data S1.** Medical history sheet.

**Data S2.** Exclusion criteria. Horses scoring ≥3 must be excluded from the study.

**Table S1.** Clinical scoring and staging system: Total score of 0 = none of the listed clinical findings. Equines with clinical signs received 1 (mild) to 3 (severe) points per finding, depending on the severity. Horses with one or more movable teeth were given 1 point. The maximum possible score was 17. ^1^Pincer‐like = large angle between the lower and upper corner incisors.

**Table S2:** Radiological scoring system. Tooth shape, tooth structure, tooth surface, and the number of teeth on which these findings were detected were evaluated. The more advanced the radiological findings, the higher the score. A maximum of 14 points could be scored.

## Data Availability

The data that support the findings of this study are openly available at https://doi.org/10.5281/zenodo.15785234.
