# Supplementary material for: Aetiological relevance of haematological, biochemical and endocrine parameters on equine odontoclastic tooth resorption and hypercementosis (EOTRH)
Source: Equine Vet J. 2025 Jul 8;58(3):699–708. doi: 10.1111/evj.14555 (PMC13041595; doi:10.1111/evj.14555)
Supplement: Supplementary file 1 — Data S1. Medical history sheet. [file EVJ-58-699-s002.pdf]

Equine Veterinary Journal BEVA

Data S1: Medical history sheet.

Medical history sheet
Please fill in for each horse.

Owner: ______________________________ **Phone number:** ______________________

Email: ______________________________

Horse: ______________________________

Identification No.: ______________________________

Gender: ☐ Mare ☐ Stallion ☐ Gelding

Pedigree:

(if possible, enclose a copy/photo of horse passport)

Age/Date of birth: ______________________

Place where bred: ☐ Germany ☐ Iceland; date of import: ________________

☐ Other: ______________________________________

Husbandry:

Current location of horse: ______________________________________

☐ Stable ☐ + pasture ☐ + paddock ☐ + paddock + pasture

☐ Open stable ☐ + pasture

☐ Pasture (24 hours)

Did a husbandry change of the horse take place?

☐ No ☐ Yes (date) ____________________________

Feeding:

Roughage: ☐ Hay ☐ Haylage ☐ Silage ☐ Hay cobs

How is the roughage fed?

☐ On the ground, horse could ingest sand

☐ On the ground, horse **COULD NOT** ingest sand

☐ Hay nets

☐ Hay racks

Concentrates: ☐ Grains ☐ Muesli ☐ Pellets

Trade name and manufacturer:

____

Feed quantity:

☐ Occasionally, e.g. after riding

☐ 100-200 g

☐ One or more kilogrammes daily

Supplements:

☐ Selenium ☐ Mushrooms

☐ Other: ____

Please state product name, quantity and duration of administration (if necessary, use page 4.)

Water supply:

☐ Tap water ☐ Well water

Does the horse have EOTRH?

☐ Not examined

☐ Yes, diagnosis was made ____________________________ *(date of diagnosis)*

Therapy:

☐ Extraction

(Please note that horses that have had all their incisors extracted cannot participate.)

☐ Selenium: ☐ Mushrooms

☐ Other: ______________________________

☐ No therapy. Regular checks by veterinarian.

Other diseases:

☐ PPID/ Equine Cushing's disease ☐ Suspected ☐ Confirmed by blood test

☐ Equine Metabolic Syndrome (EMS) ☐ Suspected ☐ Confirmed by blood test

☐ Eczema

☐ Other: ______________________________

The disease(s) has/have been known since: ______________________________

Therapy: ☐ Prascend/pergolide: _____ tablets/day

☐ Reduction in body weight

☐ Other: ______________________________

Are dental check-ups/treatments carried out regularly?

☐ No, last treatment: ______________________________ *(date of treatment)*

☐ Yes: ☐ Biannually ☐ Annually ☐ Every two years

Does the horse show any abnormalities in feed intake?

☐ No

☐ Yes: ☐ Foetor ex ore ☐ Reduced ability to grasp carrots

☐ Salivating ☐ Quidding

☐ Slow/poor feed intake

☐ Other: ______________________________

Since when? ______________________________

Other abnormalities?

☐ Behavioural changes

☐ Quiet

☐ Jumpy

☐ Head shy

☐ Loss of weight

☐ Coat change disorder

Further comments:

