# Supplementary material for: Aetiological relevance of haematological, biochemical and endocrine parameters on equine odontoclastic tooth resorption and hypercementosis (EOTRH)
Source: Equine Vet J. 2025 Jul 8;58(3):699–708. doi: 10.1111/evj.14555 (PMC13041595; doi:10.1111/evj.14555)
Supplement: Supplementary file 2 — Data S2. Exclusion criteria. Horses scoring ≥3 must be excluded from the study. [file EVJ-58-699-s004.pdf]

Equine Veterinary Journal BEVA

Data S2: Exclusion criteria. Horses scoring ≥ 3 must be excluded from the study.

**Symptom**	**Score**
**I. Behaviour**	
Calm and attentive	0
Calm	1
Apathetic	2
**II. Posture**	
Horse stands evenly on all four limbs	0
Lameness	1
Leg not bearing weight, horse lying	2
**III. Body temperature**	
37.0 – 38.0˚C	0
> 38.0˚C	2
**IV. Auscultation of the heart**	
28-48 bpm, clear, regular, well-defined heart sounds, no heart murmur	0
Tachycardia, indistinct, irregular heart murmur	2
**V. Respiratory system**	
Respiratory rate (RR): 16-26 breaths/minute (breed-specific increased RR in Icelandic horses), costoabdominal breathing type, mild inspiratory vesicular breathing sound	0
Moderate breathing sound	1
Tachypnoea, shallow breathing, wheezing	2
**Scoring**	
No stress	0
Low stress level: consult with owner, postpone further investigations to another day if necessary	1-2
Medium to high stress level: no further examinations/treatments in context of the study. Inform owner that further veterinary investigations are necessary	3-10
Termination	>3
