# Supplementary material for: Aetiological relevance of haematological, biochemical and endocrine parameters on equine odontoclastic tooth resorption and hypercementosis (EOTRH)
Source: Equine Vet J. 2025 Jul 8;58(3):699–708. doi: 10.1111/evj.14555 (PMC13041595; doi:10.1111/evj.14555)
Supplement: Supplementary file 3 — Table S1. Clinical scoring and staging system: Total score of 0 = none of the listed clinical findings. Equines with clinical signs received 1 (mild) to 3 (severe) points per finding, depending on the severity. Horses with one or more movable teeth were given 1 point. The maximum possible score was 17. 1Pincer‐like = large angle between the lower and upper corner incisors. [file EVJ-58-699-s003.pdf]

Equine Veterinary Journal BEVA

Table S1: Clinical scoring and staging system: Total score of 0 = none of the listed clinical findings.

Equines with clinical signs received 1 (mild) to 3 (severe) points per finding, depending on the severity.

Horses with one or more movable teeth were given 1 point. The maximum possible score was 17.

[1]Pincer-like = large angle between the lower and upper corner incisors.

	**Clinical findings**	**Score**
**Fistulae**	1 purulent or up to 3 serous	1
	2-3 purulent or 4-6 serous	2
	More than 3 purulent or more than 6 serous	3
**Gingival recession**	Less than 1/3 of the root exposed	1
	Less than 2/3 of the root exposed	2
	Whole root exposed	3
**Subgingival bulbous enlargement**	No	0
	Yes	1
**Calculus**	Less than 1/3 of the clinical crown covered	1
	Less than 2/3 of the clinical crown covered	2
	More than 2/3 of the clinical crown covered	3
**Gingivitis**	Focal	1
	Widespread	2
	Blueish	3
**Bite angle**	15 years and pincer-like[1]	1
	Over 15 years and bisection angle	2
	Over 15 years and pincer-like[1]	3
**Tooth mobility**	No	0
	Yes	1
**Total**		17
**Clinical Stage 0**	No clinical findings/healthy	0
**Clinical Stage 1**	Suspicious	1-2
**Clinical Stage 2**	Mild	3-5
**Clinical Stage 3**	Moderate	6-9
**Clinical Stage 4**	Severe	≥10
