# Supplementary material for: Aetiological relevance of haematological, biochemical and endocrine parameters on equine odontoclastic tooth resorption and hypercementosis (EOTRH)
Source: Equine Vet J. 2025 Jul 8;58(3):699–708. doi: 10.1111/evj.14555 (PMC13041595; doi:10.1111/evj.14555)
Supplement: Supplementary file 4 — Table S2: Radiological scoring system. Tooth shape, tooth structure, tooth surface, and the number of teeth on which these findings were detected were evaluated. The more advanced the radiological findings, the higher the score. A maximum of 14 points could be scored. [file EVJ-58-699-s001.pdf]

Equine Veterinary Journal BEVA

Table S2: Radiological scoring system. Tooth shape, tooth structure, tooth surface and the number of teeth on which these findings were detected were evaluated. The more advanced the radiological findings, the higher the scoring. A maximum of 14 points could be scored.

	**Radiological findings**	**Score**
**Quantity: teeth affected**	0	0
	1-4	1
	5-8	2
	≥9	3
**Quantity: missing/extracted teeth**	None	0
	One or more incisors already missing/extracted	1
**Tooth shape**	Regular	0
	Preserved: slightly blunted root tip, enlargement of the periodontal space	1
	Largely preserved: circumferential increase of the root tip or the more occlusal part of the tooth, intraalveolar tooth part < clinical crown	2
	Largely lost: intraalveolar tooth part = clinical crown	3
	Lost: intraalveolar tooth part > clinical crown	4
**Tooth structure**	No radiological findings	0
	Mild: single area of increased radiolucency (up to max. 1/3 of the root width)	1
	Moderate: multiple areas of increased radiolucency (up to max. 1/3) or two (up to 2/3)	2
	Severe: large areas of increased radiolucency	3
**Tooth surface**	No radiological findings	0
	1 irregularity (up to max 1/3 root length)	1
	2 irregularities/surface rough,	2
	Obviously irregular (surface indentations)/ rough	3
